# Controllable nanotopography of lysine-branched self-assembling peptide hydrogels for tendon-bone insertion regeneration

**DOI:** 10.1007/s44307-026-00111-0

**Published:** 2026-05-20

**Authors:** Xu Liu, Chenyu Wang, Xiao Zhao, Yi Xiao, Yuzhi Sun, Xin Zhang, Qingqiang Yao, Yilun Wu, Yi-shen Zhu

**Affiliations:** 1 College of Biotechnology and Pharmaceutical Engineering, Nanjing Tech University, Nanjing, 211816 China; 2 Department of Orthopaedic Surgery, Institute of Digital Medicine, Nanjing First Hospital, Nanjing Medical University, Nanjing, 210006 China; 3 Department of Orthopaedic Surgery, Nanjing First Hospital, Nanjing, 210006 China

**Keywords:** Nanotopography, Tendon-bone insertion regeneration, Lysine-branched self-assembling peptide hydrogels, Macrophage polarization, Stem cell differentiation

## Abstract

Recent advances in tissue engineering have highlighted the critical roles of both mechanical and biochemical characteristics in tendon-bone insertion (TBI) regeneration. However, the influence of surface topography, particularly the development of biocompatible hydrogels with aligned nanotopographical structures while preserving intrinsic mechanical properties, remains insufficiently explored. Here, we engineered a lysine-branched self-assembling peptide hydrogels (Lys-SAPHs) to promote TBI healing. Compared with pristine SAPH, Lys-SAPHs exhibited distinctive aligned 3D groove-like topographies without compromising mechanical integrity or hemocompatibility. In vitro, Lys-SAPHs enhanced tendon stem cells (TSCs) and bone marrow mesenchymal stem cells (BMSCs) proliferation, migration, and tendonogenic differentiation, while promoting macrophage polarization toward a reparative M2 phenotype. In a rat TBI defect model, optimized Lys-SAPH (FEK17:FEK8 = 1:6) treatment facilitated superior tendon–bone regeneration, evidenced by reduced bone defect area on micro-CT, improved Achilles functional index (AFI), and increased fibrocartilage formation compared with SAPH and control groups. This study highlighted the critical role of nanotopography in directing stem cell fate and immune modulation and presented Lys-SAPHs as a versatile platform for designing next-generation self-assembling peptide hydrogels for tendon-bone repair and broader regenerative applications.

**Supplementary Information:**

The online version contains supplementary material available at 10.1007/s44307-026-00111-0.

## Introduction

Tendon rupture is highly prevalent among individuals engaged in heavy labor and sports activities, leading to impaired joint function and reduced quality of life. For severe cases, surgical reconstruction of the tendon–bone insertion (TBI) is the standard treatment, in which the tendon is reinserted into a bone tunnel and anchored to restore load transfer, particularly in anterior cruciate ligament (ACL) and rotator cuff (RC) injuries (APostolakos et al. 2014). However, the clinical outcomes remain unsatisfactory, with reported failure rates of 10–25% for ACL reconstruction and 20–30% for RC repair (Samitier et al. 2015; Yang et al. 2023; Rodeo 2007). The poor prognosis largely arises from the limited integration at the tendon–bone interface, owing to the inherent disparity between soft and hard tissues. Moreover, the reestablishment of a functional microenvironment requires coordinated cellular responses, underscoring the importance of advanced biomaterial design to promote TBI healing and tendon function restoration.

Among the design strategies, the surface geometry of biomaterials has drawn considerable attention, particularly aligned three-dimensional (3D) topographies that recapitulate the native architecture of tissue interfaces (Du et al. 2019; Zhu et al. 2020). Such biomimetic nanotopographies provide spatial cues to regulate cell behavior and can be integrated with other tunable material properties, such as stiffness, viscoelasticity, and biochemical activity (Wang et al. 2024). In bone tissue engineering, aligned macropores and hierarchical structures have been shown to direct stem cell differentiation toward osteochondral lineages (Swanson et al. 2021; Chen et al. 2021). Likewise, topographic features can modulate the immune microenvironment by orchestrating macrophage responses. For instance, grooved or honeycomb-like patterns on titanium substrates have been demonstrated to induce macrophage elongation and promote M2 polarization, thereby enhancing tissue repair (Zhu et al. 2021; Luu et al. 2015). Despite these advances, the role of aligned 3D topography in guiding tendon regeneration and TBI reconstruction remains poorly understood, although pioneering studies suggest potential benefits (Ye et al. 2023; Dede eren et al. 2020). Furthermore, most investigations rely on rigid substrates, while fabricating aligned topographies on soft, biocompatible hydrogels—which are more suitable for mimicking native extracellular matrix (ECM)—remains a critical challenge and promising direction for TBI regenerative strategies.

Self-assembling peptide hydrogels (SAPHs) represent a class of hydrogels formed by the spontaneous organization of peptides. Inspired by the natural assembly of proteins through noncovalent interactions under mild conditions (Qi et al. 2018; Li et al. 2019a), a series of SAPHs have been developed. In our previous works, we developed an octapeptide, FEFEFKFK (FEK8), that formed a sophisticated 3D nanofibrous network via antiparallel β-sheet stacking under physiological pH conditions. The resulting biomimetic ECM system has been successfully utilized in composite scaffolds to promote osteochondral regeneration, enhance wound healing, and support the culture and maintenance of primary cells as an artificial ECM (Li et al. 2019b; Cai et al. 2022; He et al. 2022). To enhance mechanical strength, we subsequently designed an 18-amino-acid peptide composed of two tandem FEK8 motifs linked by glycine spacers, which promoted shell-like nanostructure formation (Wu et al. 2023). This observation inspired the hypothesis that the linking strategy of basic self-assembling blocks could guide the formation of aligned 3D topographies such as groove patterns.

**Scheme 1:**
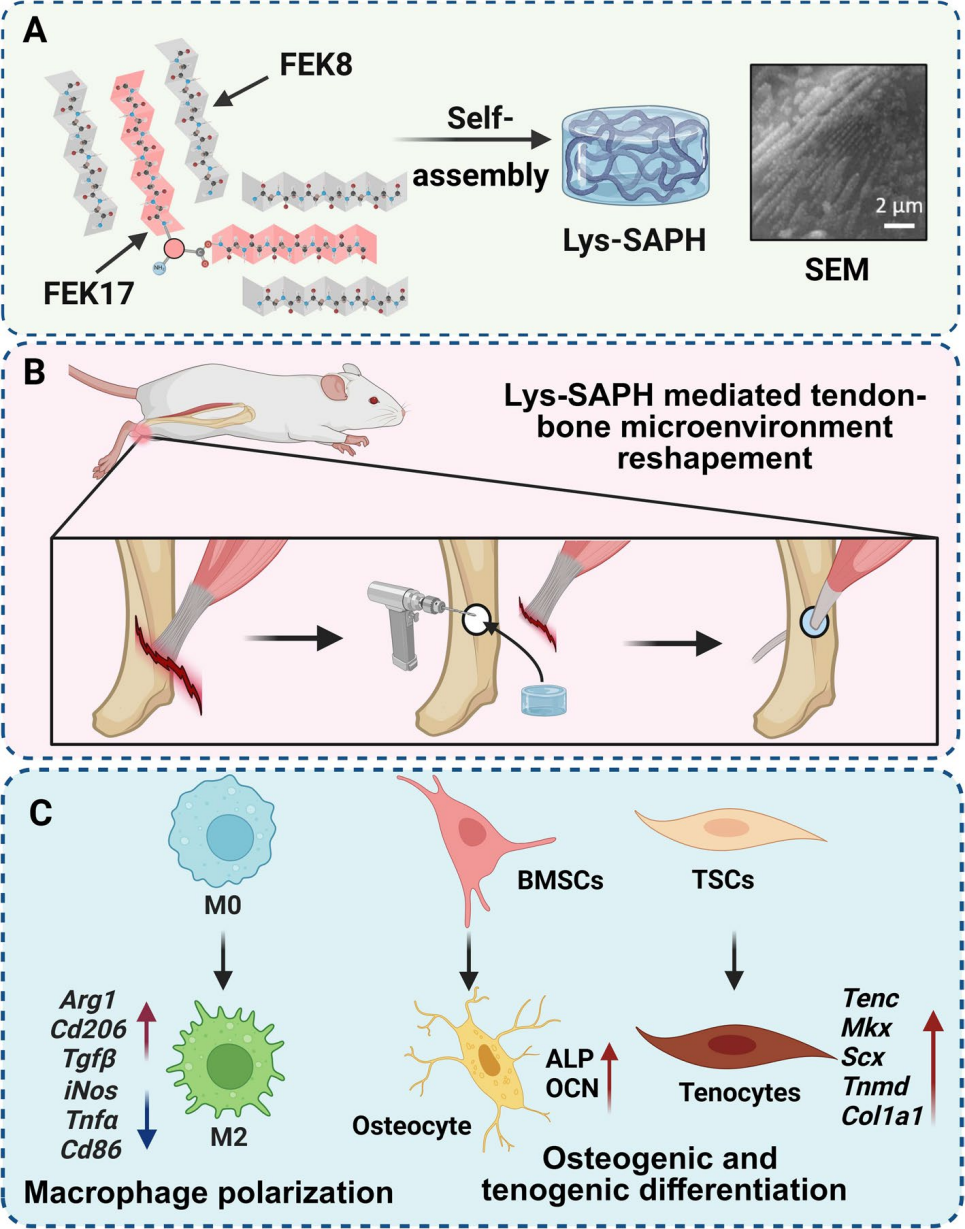
Schematic illustration of the design, fabrication, and application of a branched self-assembling peptide hydrogel (Lys-SAPH) for tendon-bone dual-defect repair. **A** Design and fabrication of Lys-SAPH and its Scanning electron microscopy (SEM) image; **B** Implantation of Lys-SAPH at tendon-bone dual-defect site in a rat model; (**C**) Proposed mechanisms by which Lys-SAPH modulates macrophage polarization toward the M2 phenotype and enhances the proliferation of tendon stem cells (TSCs) and bone marrow mesenchymal stem cells (BMSCs). Figure created with BioRender.com

Based on this rationale, we designed a lysine-branched peptide consisting of 17 amino acids, (FEFEFKFK)_2_-K (FEK17), to construct aligned 3D nanotopographies for TBI regeneration. By modulating hydrogel-forming conditions, a series of lysine-branched SAPHs (Lys-SAPHs) were obtained without chemical crosslinkers, allowing direct observation of aligned 3D topography and comparison with pristine FEK8 SAPH. RNA-sequencing was employed to elucidate the underlying mechanisms of optimized Lys-SAPH (FEK17:FEK8 = 1:6) during TBI regeneration. Furthermore, a rat tendon-bone dual-defect model was established to evaluate its reparative efficacy (Scheme 1). This strategy not only provided a novel route for constructing well-aligned 3D architectures in peptide-based or other template-free biomaterials but also demonstrated that nanotopographical control, independent of mechanical strength modulation, can effectively direct TBI regeneration.

## Materials and methods

### Materials

9-fluorenylmethyloxycarbonyl (Fmoc) protected amino acids were purchased from CSBio (Shanghai, China) for synthesis of FEK8 and FEK17 peptides (structures shown in Figure S1) using a solid phase peptide synthesis method in our lab. All the other chemical related to peptide synthesis were from Sinoreagent (China). DMEM cell culture medium (Catalog No. KGL1206-500), fetal bovine serum (FBS, Catalog No. KGA6008-50), 0.25% trypsin with EDTA (Catalog No. KGL2102-100), and all the other cell culture reagents were from Keygentec (Nanjing, China) unless specifically mentioned. Trizol, HiScript III cDNA synthesis kit, qPCR master mix, and all the other PCR reagents were from Vazyme (Nanjing, China). Antibodies (Clone code and RRID) from BioLegend (San Diego, USA): anti-mouse CD86 (GL-1, AB_313151) and anti-mouse CD206 (C068C2, AB_10901166). Anti-rat CD206 were from Proteintech (Catalog No. 18704-1-AP, RRID: AB_10597232). Anti-rat SCX were from abcam (Catalog No. ab58655, RRID: AB_882467). Water used in the experiments was deionized to Ω = 18.2 MΩ·cm.

### Preparation SAPH and Lys-SAPHs

The component of SAPH and a series of Lys-SAPHs was shown in Table 1. For each hydrogel, the peptide powders were dissolved in deionized water via vortexing, heat in an oven for 3 h at 80 °C, then adjust pH to 7.0 using NaOH (1.0 M).

**Table 1:** Nomenclature of self-assembling peptide hydrogels (SAPHs)

Name	Content
• *SAPH*	
SAPH	20 mg/mL FEK8
• *Lys-SAPH (with different molar ratio of FEK17:FEK8)*	
1:6	5 mg/mL FEK17 and 15 mg/mL FEK8, equivalent to 1:6 molar ratio
1:8	4 mg/mL FEK17 and 16 mg/mL FEK8, equivalent to 1:8 molar ratio
1:10	3.3 mg/mL FEK17 and 16.7 mg/mL FEK8, equivalent to 1:10 molar ratio
1:12	2.9 mg/mL FEK17 and 17.1 mg/mL FEK8, equivalent to 1:12 molar ratio

### Characterization

#### Transmission electron microscopy (TEM) observation

Hydrogel samples were 1:40 diluted by water with the help of vortexing, then 10 μL of sample were dropped onto a copper grid coved with 300 mesh carbon film, followed by negative staining using 2% phosphotungstic acid solution for 60 s. Then the sample was observed using a TEM (Talos L120C, Thermo, USA) at 100 kV.

#### Scanning electron microscopy (SEM) observation

One milliliter of each hydrogel sample was lyophilized, followed by gold-plating. The morphology was observed using an SEM (JSM-6510, JEOL, Japan) at 30 kV.

#### Fourier transform infrared spectrum (FTIR)

Lyophilized hydrogels were scanned using a Fourier infrared spectrometer (Evolution 201, Thermo, USA) from wavenumber 4000 to 400 cm^−1^ at a resolution 2 cm^−1^.

#### Circular dichroism (CD)

For each sample, 3 mL of diluted hydrogel were applied to a Chirascan CD spectrometer (Applied Photophysics, UK) to scan from 185 to 250 nm wavelength at a 0.5 cm path length.

#### Oscillatory rheology

For each sample, 0.5 mL of hydrogel was applied to an AR-G2 rheometer (TA instruments, USA) for recording the storage modulus (G’), loss modulus (G’’), and complex viscosity with the change of angle frequency (0.1-100 Hz) under 1% strain.

### In vitro properties of hydrogels

#### Cell culture

Rat tendon stem cells (TSCs) and rat bone marrow mesenchymal stem cells (BMSCs) were obtained from Liming Wang’ and Qingqiang Yao’ lab from Nanjing First Hospital as a gift (Sun et al n.d., Bi et al. 2007). TSCs and BMSCs were cultured in Dulbecco’s modified Eagle’s medium (DMEM) supplied with 10% fetal bovine serum (FBS) and 1% Penicillin-Streptomycin. The subculture of TSCs and BMSCs was performed using 0.25% trypsin, and experiments were carried out using cells during passage 3. RAW 264.7 cells were routinely cultured in our lab as described previously (Cheng et al. 2024). All cells were routinely tested and confirmed mycoplasma-free by PCR method.

#### In vitro compatibility and migration

##### Haemolytic assay

Fresh rat blood in a heparin tube with saline were centrifuged at 1500 rpm for 15 min to collect red blood cells (RBCs) and dispersed in saline to make a 5% RBC suspension. To test the haemolysis rate, 50 μL of RBC suspension were incubated with 140 μL of saline and 10 μL of hydrogel. Simultaneously, 50 μL of RBC incubated with 150 μL of saline and 150 μL of deionized water were prepared as negative and positive controls. After 5 h incubation at 37 °C, the supernatants of these samples were collected for recording their absorbance at 530 nm using a Synergy 2 plate reader (Biotek, USA). The haemolysis rate was calculated using the following equation:

$$\mathrm{H}\mathrm{e}\mathrm{m}\mathrm{o}\mathrm{l}\mathrm{y}\mathrm{s}\mathrm{i}\mathrm{s}\text{ } \mathrm{r}\mathrm{a}\mathrm{t}\mathrm{e} \left(\%\right)=\left({\mathrm{A}}_{\mathrm{s}\mathrm{a}\mathrm{m}\mathrm{p}\mathrm{l}\mathrm{e}}-{\mathrm{A}}_{\mathrm{n}\mathrm{e}\mathrm{g}}\right)/\left({\mathrm{A}}_{\mathrm{p}\mathrm{o}\mathrm{s}}-{\mathrm{A}}_{\mathrm{n}\mathrm{e}\mathrm{g}}\right) *100\%$$

where A_sample_, A_neg_, and A_pos_ represent the absorbance of sample, negative control, and positive control, respectively.

##### Livedead staining and CCK8 assay

TSCs and BMSCs were seeded into a 96-well plate at a density of 5000 per well. TSCs/BMSCs coculture at a ratio of 1:1 was carried out by seeding 2500 TSCs and 2500 BMSCs into each well in a 96-well plate. After overnight culture for attachment, the medium was replenished by 100 μL fresh DMEM containing 5 μL of hydrogel for further incubation of 1 to 5 days. After 3 days’ incubation, the cells were rinsed with PBS and stained with Calcein AM and propidium iodide (PI) Livedead kits (Keygentec, China), followed by observation using an XDS30 fluorescent microscope (Shunyu, China). For CCK8 assay, the culture medium was replaced by 100 μL of fresh medium containing 10 μL of CCK8 solution at each predetermined time point. After 1.5 h incubation with CCK8, the absorbance at 450 nm was measured using a Synergy 2 plate reader (Biotek, USA).

##### Wound healing assay and in vitro migration

TSCs, BMSCs, or TSCs/BMSCs co-culture at a ratio of 1:1 were seeded in a 6-well plate at 4 × 10^4^ per well. When the cell confluence reached 90%, scratches were made using a 200 μL pipette tip at the bottom of each well, followed by rinse with medium to remove the detached cells and incubated in 2 mL of serum free medium containing 100 μL of hydrogels. After incubation for 0 h, 12 h, and 24 h, the scratches were visualized using a CX23 microscope (Olympus, Japan) and analyzed using an Image J software to calculate the migration rate by area.

#### Stem cell differentiation

TSCs, BMSCs, or TSCs/BMSCs co-culture at a ratio of 1:1 were seeded in a 6-well plate at 4 × 10^4^ per well. When the cell confluence reached 70%, the culture medium was replaced by 2 mL of osteogenic-induction medium (OriCell, China) containing 100 μL of hydrogels. After 14 days, the cells were stained with alizarin red (Beyotime, China) or an alkaline phosphatase (ALP) kit (Beyotime, China) using standard protocols, followed by visualization using a CX23 microscope and analysis using an Image J software. For qPCR, the cells were harvested in Trizol reagents after 7 days’ incubation with hydrogels, followed by RNA isolation. The quality of RNA was controlled by ascertaining the A260/A280 was not less than 1.9. For each sample, 1 μg of RNA was used for the reverse transcription using a cDNA synthesis Kit (Vazyme, China) following the manufacturer’s instructions. Real-time qPCR was done for quantification of the expression of gene that related to tendonocyte differentiation. Primer sequences used were shown in Table S1.

#### Macrophage polarization

RAW 264.7 cells were seeded in a 24-well plate at a density of 5 × 10^4^ per well 1 day prior to treatment, then each well was replenished with 1 mL medium containing 50 μL of hydrogels. After 24 h’s incubation, the cells were rinsed, collected in a 1.5 mL Eppendorf tube, and stained with anti-mouse CD86 and anti-mouse CD206 antibodies (1:1000 dilution in PBS containing 2% FBS) for 15 min. Then the cells were re-suspended in antibody-free medium for analysis using a NovoCyte flow cytometer (Agilent, USA) and FlowJo software (version X.0.7). For fluorescent microscopy observation, RAW 264.7 cells were treated as above, and fixed with 4% paraformaldehyde (PFA). The staining was performed using 1:500 dilution, followed by DAPI staining (Beyotime, China) for another 5 min. Then cells were soaked in fresh PBS and visualized using an XDS30 fluorescent microscope. Real-time qPCR for RAW 264.7 cells incubated with hydrogels for 24 h were done as described in the stem cell differentiation section with primers for mouse genes in Table S1.

#### RNA-sequencing (RNA-seq)

BMSCs were seeded in a T75 culture flask at 1 × 10^6^ per well. When the cell confluence reached 70%, the culture medium was replaced by 15 mL of medium containing 750 μL of hydrogels. After 7 days, the cells were collected in Trizol reagent for RNA-seq analysis. In brief, a poly(A) mRNA capture module kit (ABclonal, China) was used to isolate mRNA, followed by library construction using a fast RNA-seq Lib Prep Kit (ABclonal, China) and a Unique Dual Index for Illumina Set_B kit (ABclonal, China). The quality was controlled by testing the distribution of peaks in the libraries using an Agilent 2100 bioanalyzer (Life Technologies, USA) according to standard protocol. For each detected gene item, the annotation was done using gene ontology (GO) and Kyoto Encyclopedia of Genes and Genomes (KEGG) database as references. Genes with fold change (FC) > 1.5 and Q value < 0.05 were considered as differentially expressed genes (DEGs). The RNA-seq and bioinformatics analysis were performed with the help of technician team from BioDeep (Suzhou, China).

### In vivo evaluation

#### Animal handling

All animal procedures were approved by the Institutional Animal Care and Use Committee (IACUC) and Animal Ethics Committee (AEC) of Nanjing First Hospital, with Approval No. IACUC-DWSY-23098402. All procedures strictly followed the "Guidelines for the Humane Treatment of Laboratory Animals" issued by National Ministry of Science and Technology. Female Sprague-Dawley (S.D.) rats (8-week-old) were purchased from Qing Long Shan Experimental Animal Co. Ltd (Nanjing, China) and randomly sorted into 4 groups according to their body weight before establishing a tendon-bone dual defect model. All animals were housing in specific pathogen-free grade (SPF) animal house, with 12 h light/dark cycle, 20-26 °C, 40-60% humidity, and allowance to regular food and water accessibility. A 1 cm incision was made on the medial of the right hind calf to expose the Achilles tendon and tibia. Then the right Achilles tendon was severed at the calcaneal of the tibia. A 1.5 mm tunnel was drilled on the distal tibia in a dorsoventrally direction. Then the Achilles tendon was inserted into the tunnel and sutured to the soft tissue for fixation. Hydrogel treatment was performed by injection of 50 μL hydrogel into the tunnel using a 1 mL syringe equipped with a 21G needle. Rats without injection postoperatively were used as control groups. All rats were treated with iodide and penicillin for infection prevention and pain control.

#### Gait analysis

The Achilles tendon function was evaluated using a CatWalk XT imaging system (Noldus, Netherland). At predetermined time points, rats walked freely across the chamber to record their behavior. For each rat, its first 3 records were eliminated from data set to avoid environmental inadaptation and stress. According to a previous report (Murrell et al. 1992), the Achilles functional index (AFI) was calculated based on the print length factor (PLF), toe spread factor(TSF), and intermediate toe spread factor (ITF):

$$\mathrm{A}\mathrm{F}\mathrm{I}=74\times \mathrm{P}\mathrm{L}\mathrm{F}+161\times \mathrm{T}\mathrm{S}\mathrm{F}+48 \mathrm{I}\mathrm{T}\mathrm{F}-5$$

$$\mathrm{P}\mathrm{L}\mathrm{F}=\left({\mathrm{P}\mathrm{r}\mathrm{i}\mathrm{n}\mathrm{t}\text{ } \mathrm{l}\mathrm{e}\mathrm{n}\mathrm{g}\mathrm{t}\mathrm{h}}_{\mathrm{L}\mathrm{e}\mathrm{f}\mathrm{t}}-{\mathrm{P}\mathrm{r}\mathrm{i}\mathrm{n}\mathrm{t}\text{ } \mathrm{l}\mathrm{e}\mathrm{n}\mathrm{g}\mathrm{t}\mathrm{h}}_{\mathrm{R}\mathrm{i}\mathrm{g}\mathrm{h}\mathrm{t}}\right)/ {\mathrm{P}\mathrm{r}\mathrm{i}\mathrm{n}\mathrm{t}\text{ } \mathrm{l}\mathrm{e}\mathrm{n}\mathrm{g}\mathrm{t}\mathrm{h}}_{\mathrm{R}\mathrm{i}\mathrm{g}\mathrm{h}\mathrm{t}}$$

$$\mathrm{T}\mathrm{S}\mathrm{F}=\left({\mathrm{T}\mathrm{o}\mathrm{e} \text{ }\mathrm{s}\mathrm{p}\mathrm{r}\mathrm{e}\mathrm{a}\mathrm{d}}_{\mathrm{R}\mathrm{i}\mathrm{g}\mathrm{h}\mathrm{t}}-{\mathrm{T}\mathrm{o}\mathrm{e}\text{ } \mathrm{s}\mathrm{p}\mathrm{r}\mathrm{e}\mathrm{a}\mathrm{d}}_{\mathrm{L}\mathrm{e}\mathrm{f}\mathrm{t}}\right)/ {\mathrm{T}\mathrm{o}\mathrm{e}\text{ } \mathrm{s}\mathrm{p}\mathrm{r}\mathrm{e}\mathrm{a}\mathrm{d}}_{\mathrm{L}\mathrm{e}\mathrm{f}\mathrm{t}}$$

$$\mathrm{I}\mathrm{T}\mathrm{F}=\left({\mathrm{I}\mathrm{n}\mathrm{t}\mathrm{e}\mathrm{r}\mathrm{m}\mathrm{e}\mathrm{d}\mathrm{i}\mathrm{a}\mathrm{t}\mathrm{e}\text{ } \mathrm{t}\mathrm{o}\mathrm{e}\text{ } \mathrm{s}\mathrm{p}\mathrm{r}\mathrm{e}\mathrm{a}\mathrm{d}}_{\mathrm{R}\mathrm{i}\mathrm{g}\mathrm{h}\mathrm{t}}-{\mathrm{I}\mathrm{n}\mathrm{t}\mathrm{e}\mathrm{r}\mathrm{m}\mathrm{e}\mathrm{d}\mathrm{i}\mathrm{a}\mathrm{t}\mathrm{e}\text{ } \mathrm{t}\mathrm{o}\mathrm{e}\text{ } \mathrm{s}\mathrm{p}\mathrm{r}\mathrm{e}\mathrm{a}\mathrm{d}}_{\mathrm{L}\mathrm{e}\mathrm{f}\mathrm{t}}\right)/ {\mathrm{I}\mathrm{n}\mathrm{t}\mathrm{e}\mathrm{r}\mathrm{m}\mathrm{e}\mathrm{d}\mathrm{i}\mathrm{a}\mathrm{t}\mathrm{e}\text{ } \mathrm{t}\mathrm{o}\mathrm{e}\text{ } \mathrm{s}\mathrm{p}\mathrm{r}\mathrm{e}\mathrm{a}\mathrm{d}}_{\mathrm{L}\mathrm{e}\mathrm{f}\mathrm{t}}$$

where the print length, toe spread, and intermediate toe spread from right (with surgery) and left (normal) hind gait were from the record system.

#### Mechanical property test

On week 8 post surgery, the rats were sacrificed to collect tibia with Achilles tendon sections. A UTM2502 hydrogel silk cloth test system (Sunstest, Shenzhen, China) was used to test the mechanical properties. By fixing the tibia and the Achilles tendon using the bottom and top clamps, the maximal load was recorded when the tendon was pulled out from the tibia tunnel.

#### Micro-CT scanning

On week 8 post surgery, the rat tibia with Achilles tendon sections were collected and scanned using a SkyScan 1176 Micro-CT (Bruker, Germany) at 70 kV voltage, 329 μA current, 1150 ms exposition, and 9 μm pixel resolution. The acquired data were re-constructed to form 3 dimensional images for analysis of bone density, bone mineral density, and bone volume/tissue volume in a cylinder (ф = 1.5 mm, height = 3 mm) centered on the geometric center of the tunnel using CTan (Version 1.6.10.1) and ImageJ software.

#### Histology analysis

On week 8 post surgery, the rat tibia with Achilles tendon sections were collected for sequentially fixing with 4% PFA, de-calcium with 10% EDTA, embedding with paraffin, and cutting into 5 μm slices. Hematoxylin & eosin (H&E) staining and Safranin red O/fast green were carried out according to the standard protocols. For immunohistological and immunofluorescent staining, the slices were treated with citrate acid (pH 6.0) buffer to recover antigen, followed by 25 min 3% H_2_O_2_ treatment to block intrinsic peroxidase. The antibody dilution in 3% BSA, followed by primary antibody staining at 4 °C overnight (1:500 dilution) and secondary antibody staining for 1 h (1:200 dilution). The immunohistological samples were treated with DAB and hematoxylin, and the immunofluorescent samples were treated with DAPI.

### Statistical analysis

All experiments were done with at least 3 biological repeats if not specifically mentioned. Data were presented as mean ± standard deviation. A GraphPad Prism software (Version 8.4.3) was used for statistical analysis. Comparison between two groups were done using One-way ANOVA with Tukey’s posttest. Statistical significance was marked with signs: ns, no significance; *, *p* < 0.05; ** *p* < 0.01; ***, *p* < 0.001; and ****, *p* < 0.0001.

## Results and discussion

### Characterization of Lys-SAPHs

**Fig. 1:**
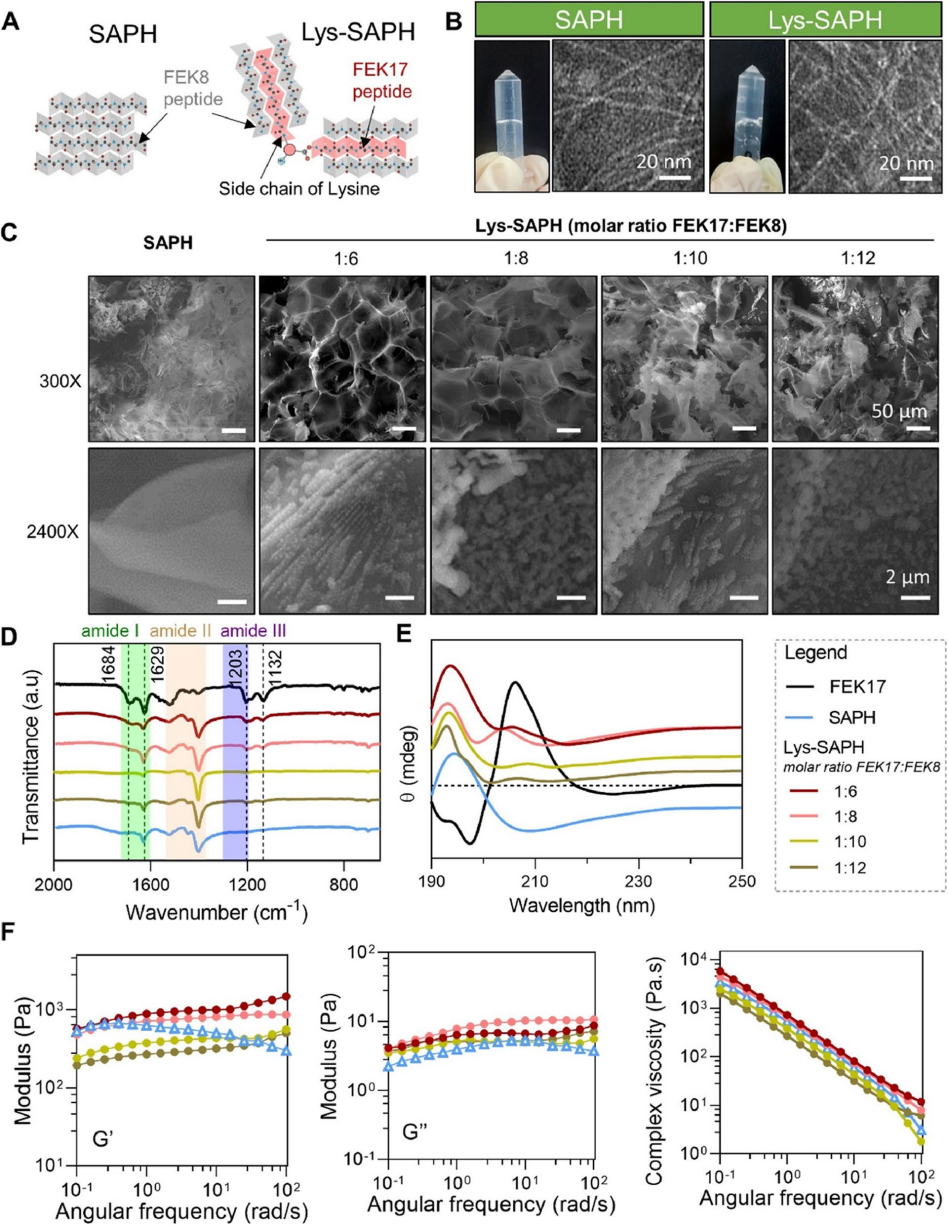
Morphology and structural characterization of SAPH and Lys-SAPHs. (**A**) Schematic illustration of SAPH and Lys-SAPHs formation. (**B**) Typical images of SAPH and Lys-SAPHs in inverted tubes and negative stained transmission electron microscope (TEM) images. (**C**) Scanning electron microscope (SEM) images illustrating the typical structure of hydrogels. (**D**) Fourier transform infrared spectroscopy and (**E**) Circular dichroism spectra of hydrogels. (**F**) Oscillating rheology of hydrogels

Based on the pristine FEK8 sequence, a lysine-branched peptide FEK17 was synthesized, in which one FEK8 unit was conjugated to the side-chain amino group of lysine (Fig. 1A). Hydrogels were fabricated using FEK8 and FEK17 (Table 1). An SAPH composed solely of FEK8 served as the control.

The resulting transparent hydrogels remained stable in inverted tubes under ambient conditions for at least seven days (Fig. 1B). TEM revealed that all hydrogels self-assembled into long, entangled nanofibers of uniform diameter (~ 2 nm) (Fig. 1C). SEM imaging further showed that the nanofibers assembled into sponge-like networks, consistent with previous observations (Wu et al. 2023; Li et al. 2019b). While SAPH displayed a smooth layer-stacked structure, Lys-SAPHs exhibited distinctive faveolated architectures, particularly pronounced at a 1:6 FEK17 to FEK8 ratio, suggesting this morphology was driven by lysine branching. Such porous architectures are considered beneficial for bone and cartilage regeneration (Li et al. 2019b) and may thus facilitate tendon-bone repair. Moreover, Lys-SAPHs revealed unique aligned 3D topographies with groove-like patterns. The 1:6 Lys-SAPH displayed the most uniform alignment, whereas pristine SAPH presented smooth, two-dimensional layers. Other FEK17:FEK8 ratios also produced groove-like topographies, though in a less ordered manner, indicating that the FEK17 fraction controls topographic formation. The underlying mechanisms driving this morphological formation warrant further structural characterization and analysis.

FTIR confirmed distinct secondary structures (Fig. 1D). SAPH exhibited an amide I peak at 1629 cm^−1^, consistent with antiparallel β-sheet formation. In contrast, Lys-SAPHs showed additional peaks at 1684 cm^−1^ (amide I) and 1203 and 1132 cm^−1^ (amide III), indicative of β-turns and altered C-N stretching/N-H bending vibrations introduced by lysine branching. CD spectroscopy corroborated these findings (Fig. 1E): FEK17 exhibits a typical β-turn structure. SAPH displayed characteristic β-sheet signals with maxima at 190 nm and minima at 210 nm, while Lys-SAPHs retained the β-sheet signature but exhibited additional features suggestive of β-turn content. Together, these results indicate that Lys-SAPH networks arise from the coordinated assembly of FEK8 and FEK17. The β-turn structures introduced by FEK17 may partially account for the formation of the groove-like topographical features observed in Lys-SAPHs.

Rheological characterization demonstrated comparable storage (G′) and loss (G′′) moduli between SAPH and Lys-SAPHs across frequencies. As in Fig. 1F, the G′ of Lys-SAPH and SAPH varied in the same order of magnitude (10^2^ to 10^3^ Pa) with frequency ranging from 10^–1^ to 10^2^ rad/s. Similarly, the corresponding G′′ of the tested hydrogels were from 10^0^ to 10^1^ Pa. Consequentially, the complex viscosity of Lys-SAPHs increased slightly in a FEK17 dose-dependent manner, reflecting a weak enhancement in mechanical robustness upon incorporation of lysine-branch peptides (Wu et al. 2023). Given the relatively small differences in complex viscosity among the hydrogels, the variations in topology were not easily manifested at the macroscopic mechanical level.

### In vitro properties of hydrogels

#### Effect of hydrogels on stem cell performance

**Fig. 2:**
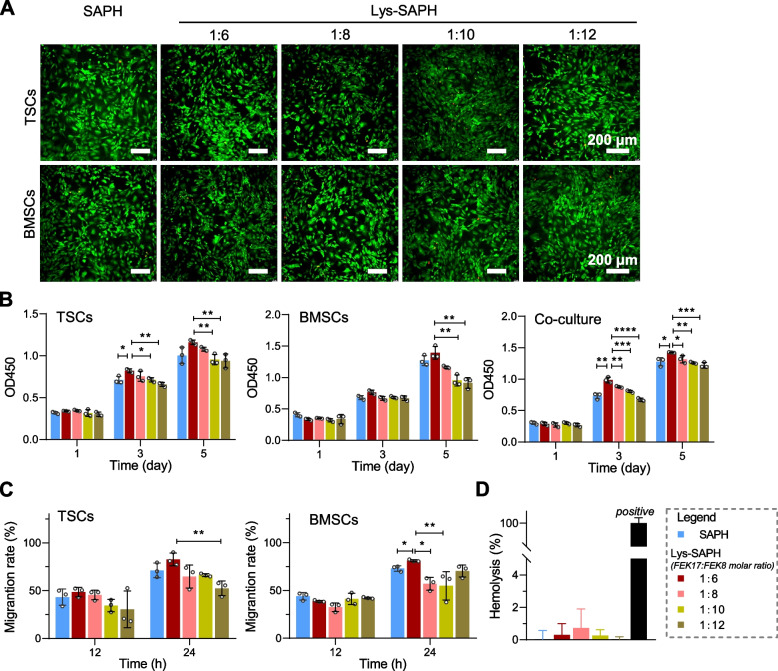
Effect of SAPH and Lys-SAPHs on tendon stem cells (TSCs) and bone marrow mesenchymal stem cells (BMSCs). (**A**) Live-dead staining of TSCs and BMSCs incubated with hydrogels for 3 days. (**B**) The optical density values at 450 nm (OD_450_). (**C**) The in vitro migration potency assessed by wound healing assay. (**D**) Hemolysis rate of hydrogels. Data are presented as mean ± SD (n = 3). * p < 0.05; ** *p* < 0.01; ***, *p* < 0.001; and ****, *p* < 0.0001

We next investigated whether the Lys-SAPHs could impart distinct cellular responses. During tendon–bone healing, TSCs and BMSCs are essential contributors. Biomaterials designed for this application must therefore support TSCs and BMSCs recruitment, proliferation, and lineage-specific differentiation. Accordingly, we examined the in vitro behaviors of TSCs and BMSCs cultured with SAPH or Lys-SAPHs. After three days of incubation, neither hydrogel induced significant cell death, and no obvious morphological changes were observed (Fig. 2A).

Cell proliferation was assessed in TSCs, BMSCs, and TSCs/BMSCs co-cultures. Lys-SAPHs elicited an FEK17 dose-dependent increase in proliferation, with 1:6 ratio producing the highest OD_450_ values at both day 3 and day 5 (Fig. 2B). This indicates that Lys-SAPHs provide a favorable microenvironment for stem cell proliferation, with 1:6 ratio exhibiting the highest proliferative capacity. This optimal ratio appears to provide the most suitable ECM-like environment for cellular growth, thereby facilitating tendon repair. A wound healing assay further demonstrated that TSCs and BMSCs migration rates were comparable between SAPH and Lys-SAPH with 1:6 ratio but decreased in Lys-SAPHs with 1:8, 1:10, and 1:12 ratios (Fig. 2C and Figure S2). This trend paralleled differences in complex viscosity, indicating that hydrogel toughness and its faveolated architectures with appropriately sized pores jointly contribute to stem cell migratory capacity (López-serrano et al. 2025). All hydrogels exhibited < 5% hemolysis (Fig. 2D), suggesting these hydrogels did not exhibit a significant tendency toward erythrocyte swelling.

Among all tested formulations, Lys-SAPH with 1:6 ratio displayed the most distinct and well-aligned 3D nanotopography, along with comparable mechanical properties and hemolysis rates to those of pristine SAPH. Therefore, this formulation (FEK17:FEK8 = 1:6) was selected for subsequent biological evaluations and is hereafter referred to simply as Lys-SAPH.

#### Stem cell differentiation of hydrogels

**Fig. 3:**
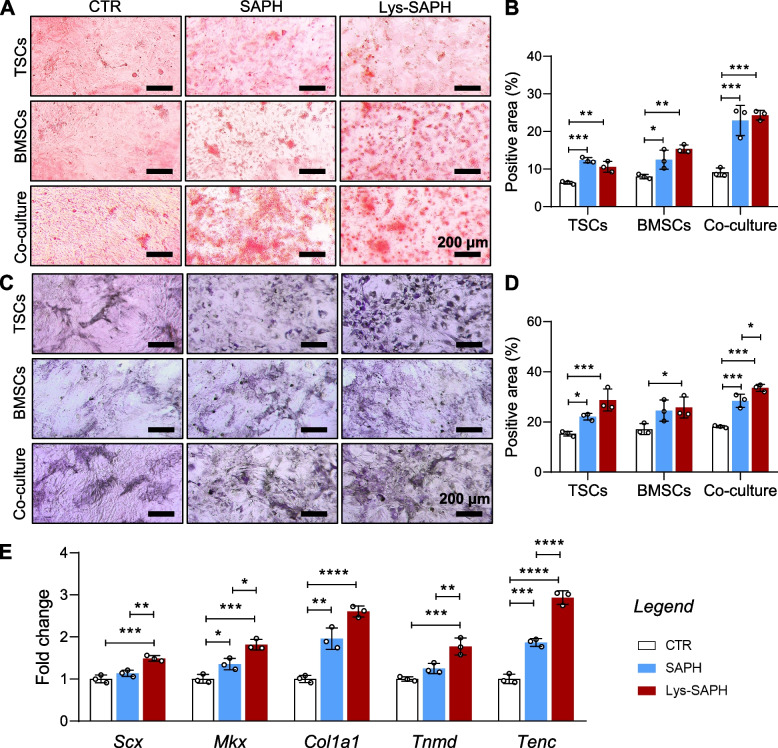
Effect of hydrogels on stem cell differentiation. (**A**, **B**) Alizarin red staining and (**C**, **D**) alkaline phosphatase (ALP) activity staining of TSCs, BMSCs, and TSCs/BMSCs co-culture incubated with hydrogels for 14 days. (**E**) The mRNA expression fold changes of TSCs incubated with different hydrogels for 7 days. Data are presented as mean ± SD (n = 3). * p < 0.05; ** *p* < 0.01; ***, *p* < 0.001; and ****, *p* < 0.0001

To assess osteogenic differentiation, alizarin red and ALP staining were performed. TSCs, BMSCs, and co-cultures exhibited robust calcium deposition and increased ALP activity when cultured with hydrogels (Fig. 3A-D). Notably, Lys-SAPH induced greater ALP-positive staining in TSCs and co-cultures compared to SAPH, suggesting that aligned topography may enhance TSCs-mediated osteogenesis. Tendon-related differentiation was examined by qPCR analysis of *Scx*, *Mkx*, *Col1a1*, *Tnmd*, and *Tenc* expression in TSCs (Fig. 3E). All hydrogels promoted tendonogenic gene expression, with Lys-SAPH eliciting higher levels than SAPH. Given the similar bulk mechanical properties, these enhancements were likely attributable to lysine branching and the aligned 3D topography. Moreover, the chemical changes from the lysine branching (e.g., the density of immobilized charge, intramolecular volume) could also be a factor(Shavykin et al. 2020).

These findings suggested that the enhanced osteogenic and tenogenic differentiation induced by Lys-SAPH raised from its biochemical and topographical cues rather than stiffness differences.

#### Hydrogel-regulated macrophage-related immune microenvironment for tendon-bone healing

**Fig. 4:**
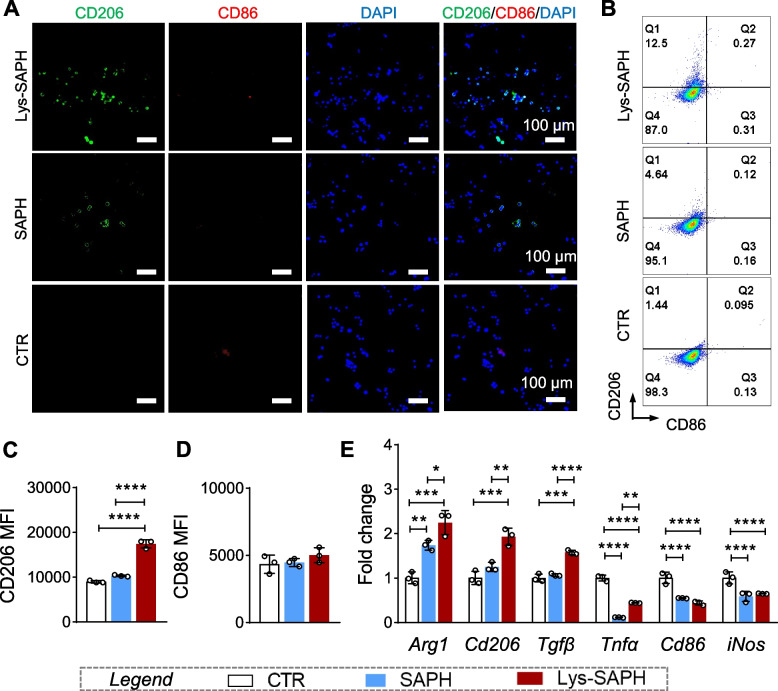
The potency of hydrogels to reshape the immune microenvironment by induction of macrophage polarization. (**A**) Fluorescent microscopy images of RAW 264.7 cells incubated with hydrogels for 24 h. (**B**) Pseudo colored dot plots and mean fluorescent intensity (MFI) of (**C**) CD206 and (**D**) CD86 of RAW 264.7 cells detected by flow cytometry after 24 h incubation. (**E**) The mRNA expression fold changes. Data are presented as mean ± SD (n = 3). * p < 0.05; ** *p* < 0.01; ***, *p* < 0.001; and ****, *p* < 0.0001

The interaction between biomaterials and the local immune microenvironment plays a pivotal role in tissue regeneration. Among the regulatory mechanisms, macrophage polarization has emerged as a central mediator. To evaluate the immunomodulatory capacity of Lys-SAPH, RAW264.7 cells were incubated with SAPH or Lys-SAPH. Immunofluorescence analysis revealed that CD206 expression was markedly increased following hydrogel treatment, whereas CD86 expression remained negligible (Fig. 4A). These results indicated that both SAPH and Lys-SAPH favored M2-like macrophage polarization, which was conducive to tissue repair.

Flow cytometry further confirmed these observations (Fig. 4B). While CD86 (M1 marker) levels were unchanged across groups (Fig. 4D), Lys-SAPH-treated cells exhibited a 1.7-fold increase in CD206 mean fluorescence intensity (MFI) compared with SAPH-treated cells (Fig. 4C), suggesting the unique contribution of aligned 3D topography in promoting M2 polarization. In all cases, the CD86 MFIs were maintained during treatment. We further analyzed the gene expression related to macrophage polarization. As in Fig. 4E, Gene expression analysis provided additional evidence: Lys-SAPH upregulated M2-associated genes (*Arg1*, *Cd206*, and *Tgfβ*) while downregulating M1-associated genes (*Tnfα*, *Cd86*, and *iNos*). Despite the lack of further validation on the protein levels and function, the above data still showed a clear trend of Lys-SAPH induced macrophage polarization.

Macrophage polarization from a pro-inflammatory M1 phenotype to a reparative M2 phenotype played a pivotal role in tendon–bone healing. In the early inflammatory phase, M1 macrophages mediated host defense by releasing pro-inflammatory cytokines such as TNF-α; however, their prolonged activation might result in chronic inflammation and fibrotic tissue formation, ultimately hindering tendon–bone integration (Millar et al. 2017). In contrast, M2 macrophages secreted anti-inflammatory cytokines (e.g., TGF-β1) and various growth factors that regulated ECM remodeling, angiogenesis, and fibroblast differentiation into tenocytes (Chen et al. 2025). Furthermore, M2 macrophages facilitated type I collagen deposition, promoted the organization of Sharpey-like fibers, and supported endochondral ossification at the tendon-bone interface (Jang et al. 2015).

Collectively, these findings suggested that Lys-SAPH actively modulated the local immune microenvironment by promoting macrophage polarization toward the M2 phenotype. This immunoregulatory shift contributed to the resolution of inflammation and initiated matrix-mediated tissue remodeling, thereby establishing a regenerative milieu conducive to tendon-bone healing. Such immunomodulatory activity might contribute to the accelerated ECM remodeling, enhanced collagen alignment, and improved maturation of regenerated tendon tissue observed in vivo, highlighting the mechanistic interplay between immune regulation and functional tendon–bone integration.

#### RNA-seq analysis of hydrogels

**Fig. 5:**
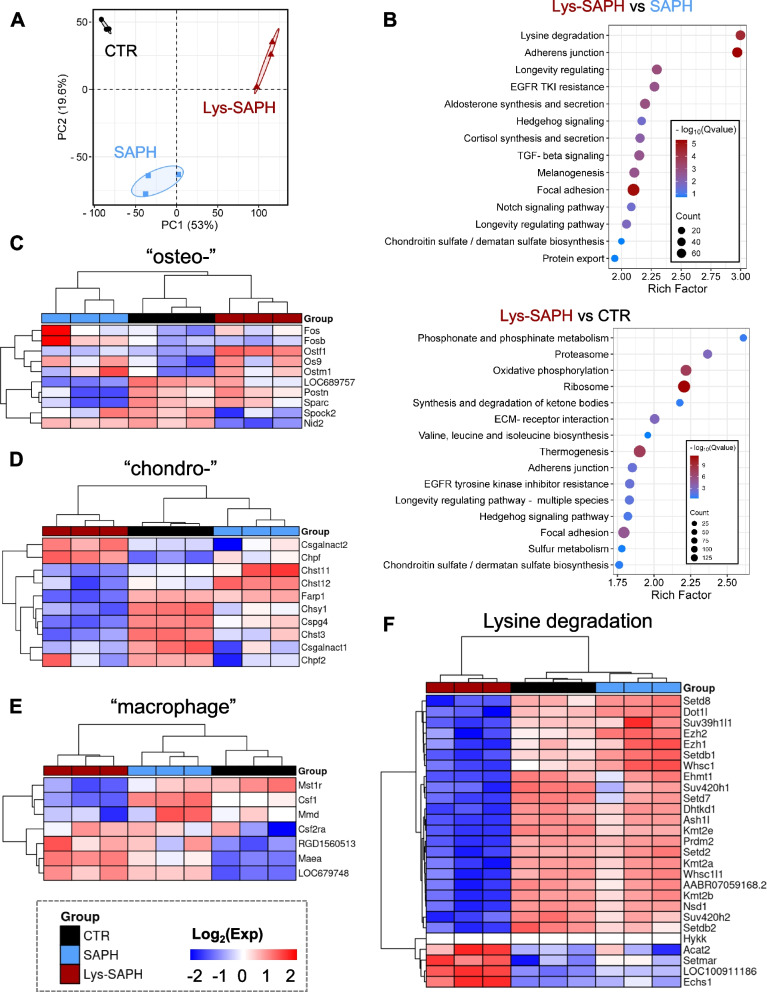
RNA sequencing (RNA-seq) analysis in rat BMSCs incubated with corresponding hydrogels for 7 days. (**A**) The plot of principal component analysis (PCA) with confidence ellipse. (**B**) The top 15 KEGG pathways significantly enriched for the differentially expressed genes (DEGs) between Lys-SAPH vs SAPH and Lys-SAPH vs CTR. Clustering heatmaps of DEGs correlated to (**C**) osteogenesis, (**D**) chondrogenesis, (**E**) macrophage, and (**F**) lysine degradation

RNA sequencing analysis provided mechanistic insights into how Lys-SAPH modulated the biological behavior of BMSCs compared with SAPH and the untreated control (CTR). Principal component analysis (PCA) revealed distinct clustering of the three groups, confirming that Lys-SAPH exerted unique transcriptional regulation on stem cells (Fig. 5A).

KEGG pathway enrichment analysis demonstrated that Lys-SAPH markedly influenced ECM-receptor interactions, focal adhesion, and glycosaminoglycan metabolism, suggesting enhanced cell-matrix communication and niche remodeling (Fig. 5B). Analysis of osteogenesis-related genes suggested that Lys-SAPH and SAPH similarly supported osteogenic processes, as reflected by the expression of *Sparc*, *Spock2*, and *Nid2* (Fig. 5C). However, Lys-SAPH uniquely upregulated genes related to osteoclast function (*Ostf1*, *Postn*, *LOC689757*) while downregulating pathological osteopetrosis (*Ostm1*) and osteosarcoma-related oncogenes (*Os9, Fos, Fosb*). Chondrogenesis-related genes (*Csgalnact2*, *Chpf*, *Chst11*, and *Chst12*) were also upregulated in both SAPH and Lys-SAPH groups (Fig. 5D). These genes, which regulated chondroitin sulphate biosynthesis, were critical for enhancing tendon–bone healing by supporting hyaline cartilage formation and reducing capillary infiltration (Taşkesen et al. 2015). Interestingly, *Csgalnact2* and *Chpf* were also implicated in macrophage regulation, with higher expression under Lys-SAPH treatment.

These results indicated a coordinated promotion of osteogenesis, osteoclastogenesis and chondrogenesis while maintaining physiological bone turnover.

Interestingly, Lys-SAPH also exhibited a pronounced immunomodulatory signature (Fig. 5E). It downregulated macrophage activation and differentiation genes (*Mst1r*, *Csf1*, *Mmd*), while enhancing expression of genes (*RGD1559921*, *LOC679748*) associated with restricted macrophage migration. This transcriptional profile supporteed the in vitro and in vivo findings that Lys-SAPH fostered a reparative, anti-inflammatory microenvironment conducive to tendon-bone integration.

In addition, genes associated with the lysine degradation pathway were broadly downregulated in the Lys-SAPH group (Fig. 5F). This transcriptional change should not be construed as evidence of direct intracellular metabolism of lysine derived from the hydrogel, as peptide-bound lysine is unlikely to enter canonical cellular degradation pathways without intracellular processing. Instead, it may reflect an indirect regulatory effect of lysine side-chain modification on the local microenvironment. Such modification may enhance matrix stability and alter extracellular biochemical cues, thereby influencing cellular metabolic programs at the transcriptional level. Alternatively, extracellular processing of lysine-containing peptide segments may modulate local amino acid-related signaling. These effects collectively suggested that Lys-SAPH influenced metabolic gene expression through microenvironmental regulation rather than direct metabolic incorporation.

Overall, the transcriptomic analysis further corroborated the findings from stem cell and macrophage experiments, underscoring the multifunctional role of Lys-SAPH in the healing process. Specifically, Lys-SAPH simultaneously promoted osteochondral differentiation, mitigated macrophage-mediated inflammatory responses, and enhanced ECM organization and maturation, thereby providing a molecular basis for its superior efficacy in tendon-bone healing.

### The in vivo evaluation of tendon–bone interface (TBI) regeneration

**Fig. 6:**
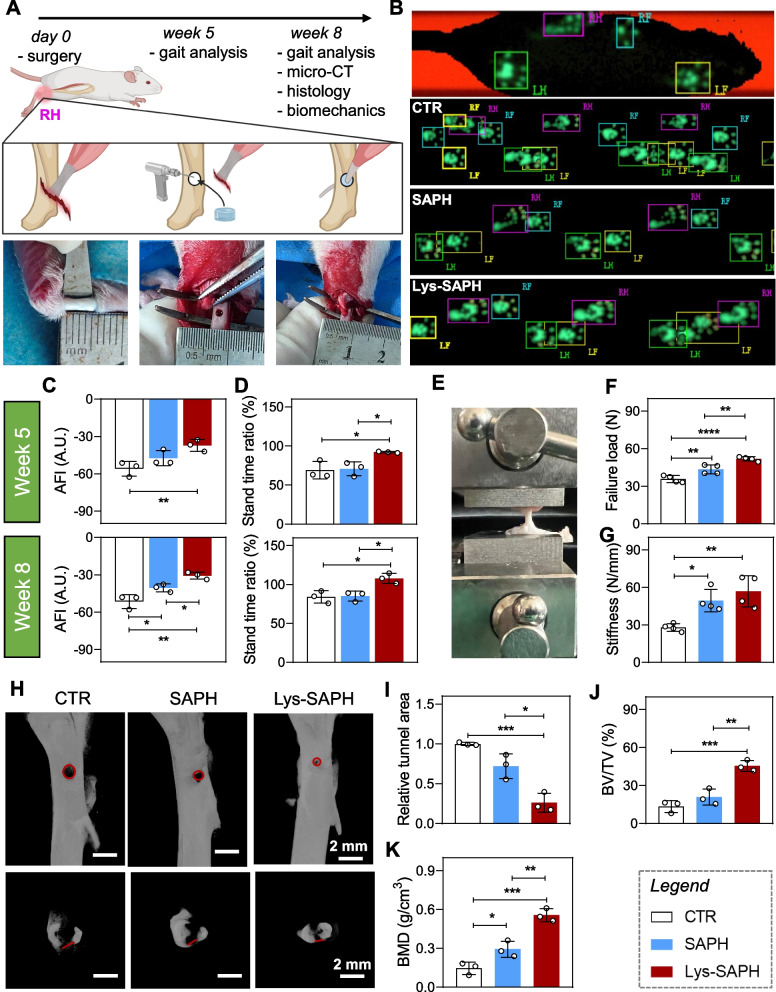
The functional and mechanical properties of TBI recovery. (**A**) Schematically illustration of the establishment of a tendon-bone defect model on the right hind (RH) leg near Achilles joint and proposed data collection schedule, with images indicating the corresponding process. Figure created with BioRender.com. (**B**) Annotation of rat footprints and representative footprint records from each group on week 8 post surgery. Histograms showing the statistics of (**C**) Achilles functional index (AFI) and (**D**) Stand time ratio. (**E**) An image showing an Achilles tendon loaded onto a hydrogel silk cloth test system. (**F**) Maximal failure load and (**G**) stiffness of the tendon after 8 week’s recovery with different groups. (**H)** The reconstructed micro-CT images showing the coronal and transactional planes of rat tibia at week 8 post surgery, with red lines indicating the boundary of defected bone. (**I**, **J**, **K**) Histograms of relative tunnel area, bone volume/tissue volume (BV/TV) ratio, and bone mineral density (BMD). Data are presented as mean ± SD (n = 3). * p < 0.05; ** *p* < 0.01; ***, *p* < 0.001; and ****, *p* < 0.0001

A rat tendon–bone dual-defect model was established to assess the regenerative efficacy of Lys-SAPH in vivo (Ni et al. 2023). Briefly, the right Achilles tendon was severed at its calcaneal insertion, implanted into a hydrogel-infilled tibial drill hole, and sutured to adjacent soft tissue for fixation (Fig. 6A). Rats without hydrogel treatment after surgery were used as a control (CTR).

#### Gait analysis

Functional recovery was first evaluated by gait analysis. At 8 weeks post-surgery, CTR group of animals exhibited larger, fainter right hind (RH) footprints compared to the left hind (LH), whereas Lys-SAPH-treated rats displayed longer step lengths and firmer RH footprints (Fig. 6B). Quantitative analysis demonstrated significant improvements in the AFI at weeks 5 (–37.2) and 8 (–30.6) compared with CTR (–55.9, –51.5) and SAPH (−47.1, –40.6) (Fig. 6C). Similarly, stand time ratios confirmed superior functional restoration in Lys-SAPH group (Fig. 6D).

#### Mechanical property test

Biomechanical tests at 8 weeks further supported these findings. Tendons from Lys-SAPH group achieved the highest failure load (51.8 N) compared to CTR (35.7 N) and SAPH (43.5 N) groups, as well as the greatest stiffness among all groups (Fig. 6E-G), demonstrating enhanced mechanical competence.

#### Micro-CT scanning

Bone regeneration within the tibial defect was examined by micro-CT. Representative scans highlighted reduced tunnel areas in Lys-SAPH group relative to SAPH and CTR (Fig. 6H-I). Quantitative analysis revealed that Lys-SAPH treatment yielded the highest bone volume/tissue volume (BV/TV) ratio (45.5%), equivalent to 3.4-fold and 2.2-fold increases over CTR and SAPH, respectively (Fig. 6J). Consistently, bone mineral density (BMD) was significantly elevated in Lys-SAPH group (0.56 g/cm^3^), representing 3.8-fold and 1.9-fold increases relative to CTR and SAPH (Fig. 6K).

These findings demonstrated that Lys-SAPH substantially enhanced tendon functional recovery and bone tissue formation, highlighting the potential role of their aligned 3D nanotopography in promoting tendon–bone integration.

#### Histology analysis of tendon-bone interfaces

**Fig. 7:**
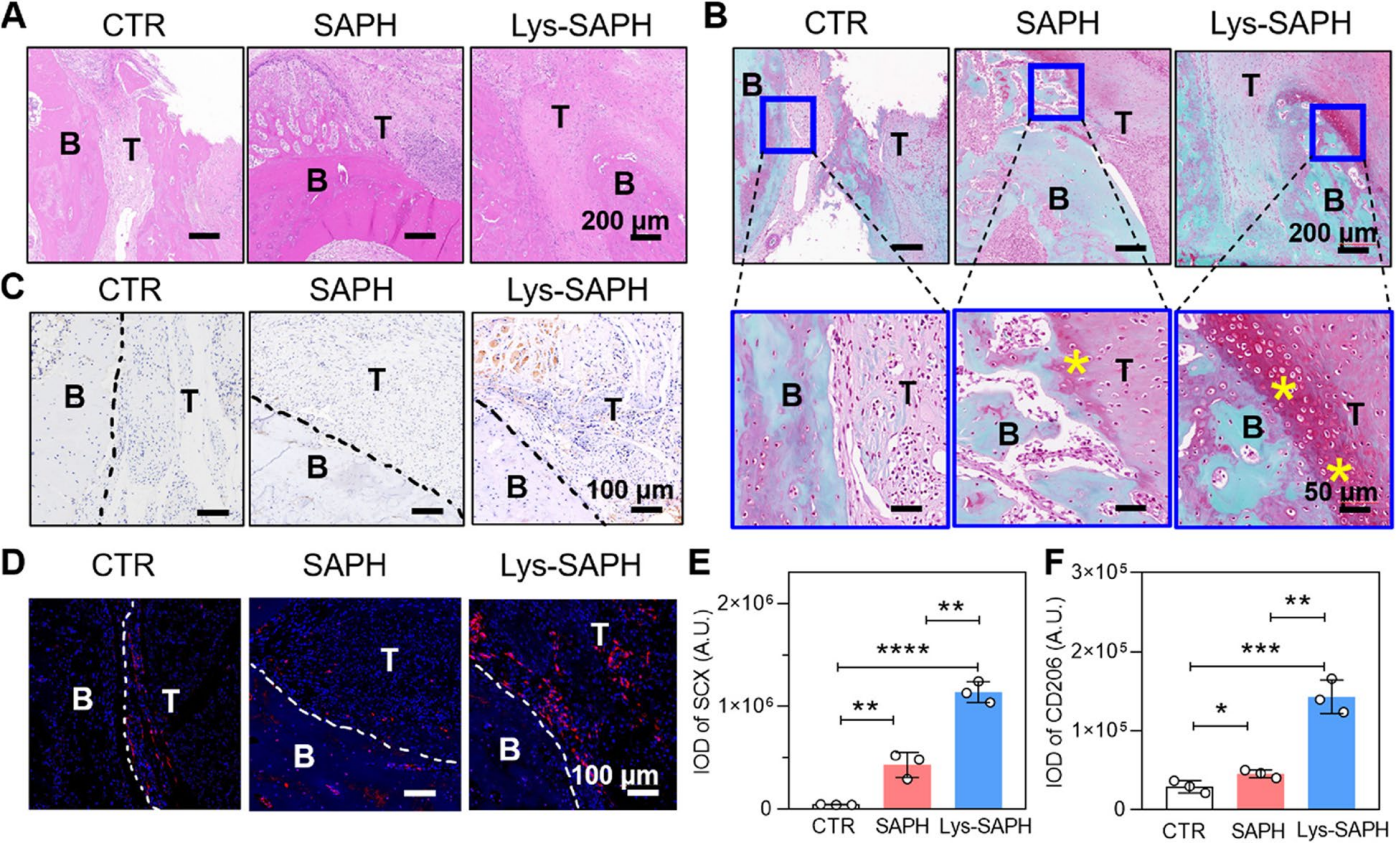
Histology analysis of the Tendon-Bone Interface (TBI). (**A**, **B**, **C**, **D**) Typical images of defected tendon-bone at week 8 post surgery stained with Hematoxylin and Eosin (H&E), safranin O/fast green, Scleraxis (SCX), and CD206/DAPI. (**E**, **F**) Histograms showing the integrated option density (IOD) values of SCX and CD206. Labels on slices: “B” for bone tissue, “T” for tendon tissue, “* in yellow” for fibrocartilage. Data are presented as mean ± SD (n = 3). * p < 0.05; ** *p* < 0.01; ***, *p* < 0.001; and ****, *p* < 0.0001

To evaluate tendon–bone interface formation, histological examinations were performed on harvested tissues at 8 weeks post-surgery using hematoxylin and eosin (H&E) and safranin O/fast green staining. In H&E sections, bone appeared as dense dark pink areas (“B”), while tendon was identified as lighter, hollow pink regions (“T”) (Fig. 7A). As expected, the tendon was anchored within the bone tunnel, confirming successful model establishment. Lys-SAPH group exhibited a more continuous and organized transition zone, characterized by densely aligned collagen fibers and reduced inflammatory cell infiltration compared with CTR. In contrast, CTR group displayed irregular tissue organization and numerous inflammatory cells at the interface, suggesting delayed healing and poor integration. In addition, no significant immune-related adverse effects were observed in the H&E slices, suggesting the biosafety of our hydrogels. Indeed, we implanted similar hydrogels without Lys-branch into rabbits with segmental bone defects for as long as 6 months, and the results suggested the long-term biosafety of these peptide hydrogels (Wu et al. 2023). When rats were sacrificed on Week 8, all hydrogels were degraded visually, suggesting the peptide hydrogels are metabolicable in vivo.

The formation of fibrocartilage sandwich layers at the tendon-bone interface was widely recognized as a hallmark of functional enthesis regeneration, as these layers facilitated gradual stress transfer and enhanced load-bearing capacity. Safranin O/fast green staining revealed fibrocartilage formation in both SAPH and Lys-SAPH groups (Fig. 7B). Notably, Lys-SAPH treatment resulted in the thickest and best-organized fibrocartilage layer, whereas CTR group exhibited direct tendon-bone contact without fibrocartilage, a configuration associated with impaired repair outcomes. Consistent with these observations, Sharpey-like fibers bridging tendon and bone were observed in both SAPH and Lys-SAPH groups (indicated by yellow asterisks in Fig. 7B, lower panels), but were absent in CTR group. These fibers appeared as green-stained, collagen-rich bundles inserted into the bone matrix, with Lys-SAPH group displaying a higher density and more uniform alignment. This structural reinforcement was in agreement with transcriptomic enrichment of ECM-receptor interaction and chondroitin sulfate biosynthesis pathways, which were known to support fibrocartilage maturation and interface stability.

To further assess tenogenic differentiation, scleraxis (SCX), an early tendon lineage marker, was evaluated by immunostaining. Quantitative analysis revealed a clear trend of SCX expression in the order of Lys-SAPH > SAPH > CTR (Fig. 7C, 7E), indicating enhanced tenocyte differentiation induced by the lysine-branched nanotopography. This finding is consistent with the RNA-seq data showing upregulation of tendon- and ECM-related genes under Lys-SAPH treatment.

In parallel, macrophage phenotype distribution at the tendon–bone interface was examined by CD206 immunofluorescence staining. CD206^+^ M2-like macrophages were predominantly observed along the tendon-bone junction and within the fibrocartilage layer in Lys-SAPH group (Fig. 7D), whereas SAPH and CTR groups exhibited only sparse and peripheral CD206^+^ signals. Quantitative analysis revealed the highest CD206 expression in Lys-SAPH group (Fig. 7F). This spatially localized enrichment of M2 macrophages correlated with transcriptomic suppression of macrophage activation and migration-related genes and supported the establishment of an anti-inflammatory, matrix-remodeling microenvironment conducive to tendon-bone healing.

Together, these in vivo findings demonstrated that Lys-SAPH promoted a structurally and biologically integrated tendon-bone interface, characterized by robust fibrocartilage formation, enhanced tenogenic differentiation, and targeted enrichment of M2 macrophages. When considered alongside the transcriptomic data, the results indicated that Lys-SAPH drove tendon-bone regeneration through coordinated regulation of osteochondral differentiation, ECM remodeling, and immune modulation, ultimately facilitating functional interface maturation.

## Conclusion

In this study, we developed lysine-branched self-assembling peptide hydrogels (Lys-SAPHs) as a novel biomaterial for TBI regeneration. Lys-SAPHs exhibited distinctive aligned nanotopographical architectures with enhanced viscoelasticity and favorable hemocompatibility. Functionally, Lys-SAPHs dose-dependently promoted TSCs and BMSCs proliferation, migration, and osteogenic differentiation, while simultaneously directing macrophage polarization toward a reparative M2 phenotype. Transcriptomic analysis revealed that optimized Lys-SAPH (FEK17:FEK8 = 1:6) modulated multiple biological pathways, including osteogenesis, osteoclastogenesis, chondrogenesis, macrophage differentiation and migration, as well as lysine metabolism. In a rat TBI dual-defect model, Lys-SAPH significantly enhanced bone formation, fibrocartilage layer development, and tendon functional recovery. Overall, these findings established Lys-SAPHs as a versatile platform that integrated structural, mechanical, and immunomodulatory advantages, combining self-assembly with branched peptides, thereby offering strong potential for tendon-bone repair and broader applications in regenerative medicine.

## Supplementary Information

Supplementary Material 1

## Data Availability

RNA-Seq data were deposited into the Sequence Read Archive (SRA) under accession number PRJNA1166140 and are available at the following URL: https://www.ncbi.nlm.nih.gov/sra/PRJNA1166140.
