# Supplementary material for: Controllable nanotopography of lysine-branched self-assembling peptide hydrogels for tendon-bone insertion regeneration
Source: Adv Biotechnol (Singap). 2026 May 20;4(2):22. doi: 10.1007/s44307-026-00111-0 (PMC13190967; doi:10.1007/s44307-026-00111-0)
Supplement: Supplementary file 1 — Supplementary Material 1 [file 44307_2026_111_MOESM1_ESM.docx]

**Controllable Nanotopography of Lysine-branched Self-assembling Peptide Hydrogels for Tendon-Bone Insertion Regeneration**

Xu Liu,^1^ Chenyu Wang,^1^ Xiao Zhao,^2^ Yuzhi Sun,^2^ Xin Zhang,^3^ Qingqiang Yao,^2^ Yilun Wu,^1^* Yi-shen Zhu^1^*

*Corresponding authors

College of Biotechnology and Pharmaceutical Engineering, Nanjing Tech University, Nanjing 211816, China

^2^ Department of Orthopaedic Surgery, Institute of Digital Medicine, Nanjing First Hospital, Nanjing Medical University, 210006, Nanjing, China

^3^ Department of Orthopaedic Surgery, Nanjing First Hospital, 210006, Nanjing, China

E-mail: zhuyish@njtech.edu.cn (Y.Z.); wuyilun@njtech.edu.cn (Y.W.);

Tel: +86-18900660563


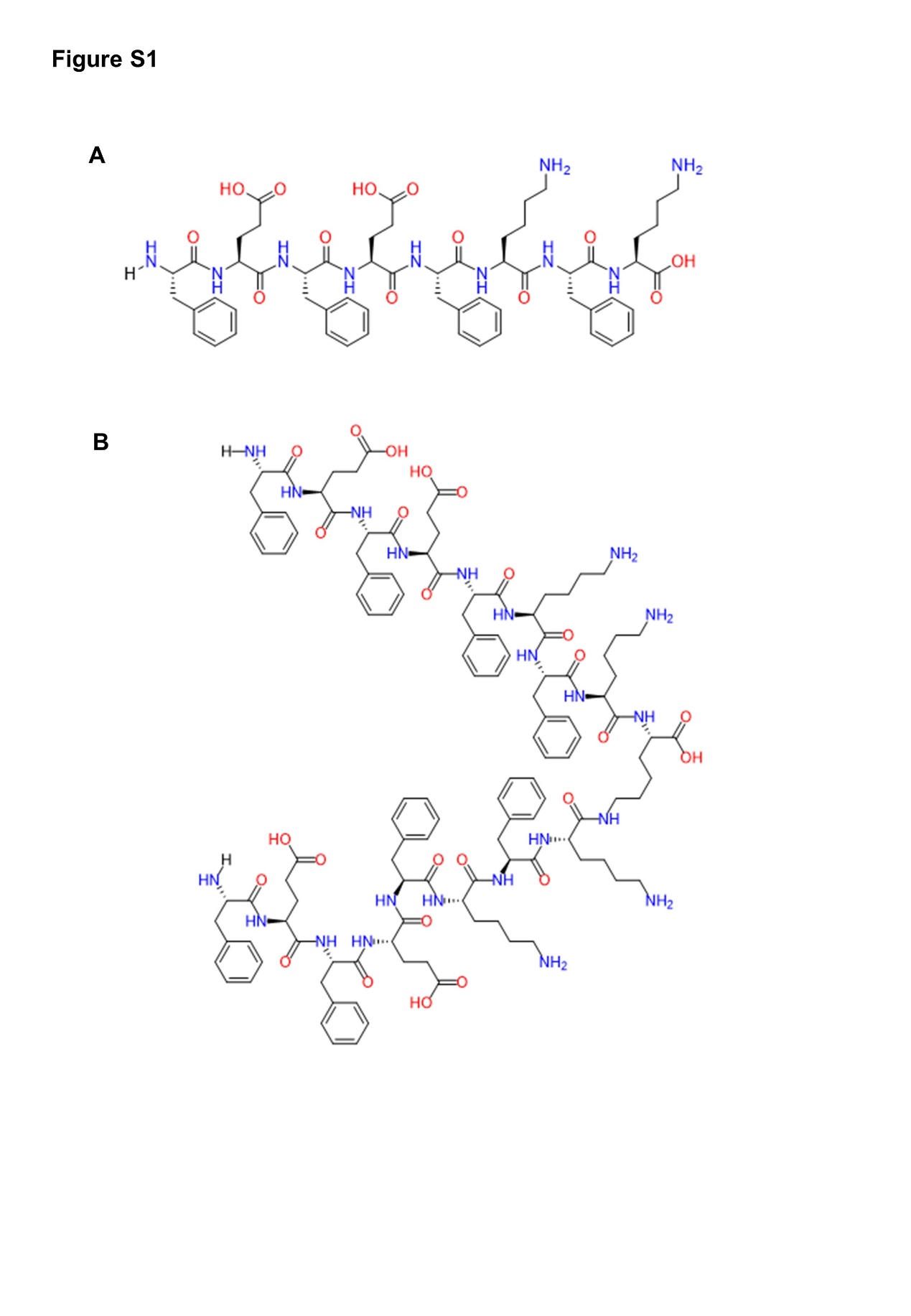


**Figure S1 The chemical structures of FEK8 and FEK17 peptides.**

(A) FEK8 peptide with a sequence of FEFEFKFK; (B) FEK17 peptide with a sequence of (FEFEFKFK)_2_-K


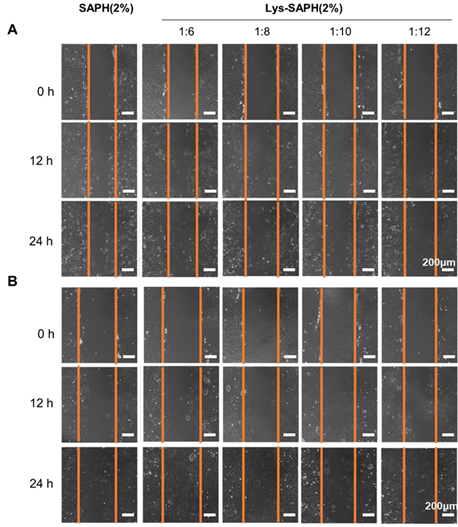


**Figure S2.** Typical images of (A) TSCs and (B) BMSCs in wound healing assay at 0h, 12h, and 24h.

**Table S1 List of primer sequences**

| Genes |  | Primer sequences (5’-3’) |
| --- | --- | --- |
| - Rat genes for TSCs differentiation | | |
| *Scx* | Forward  Reverse | TCATCCCGACCGAGCCAGCA  CCGCAGGCTTCACCCACCAG |
| *Mkx* | Forward  Reverse | TTTACAAGCACCGTGACAACCC  ACAGTGTTCTTCAGCCGTCGTC |
| *Tnmd* | Forward  Reverse | TGGAAATGGCACCGATGAAAC  GCAGGAACCCAAATCACTGACTG |
| *Tenc* | Forward  Reverse | CAGAAGCTGAACCGGAAGTTG  GGCTGTTGTTGCTATGGCGCT |
| *Col1a1* | Forward  Reverse | CTCCTGGCAAGAACGGAGATGA  CTCCTTTGGCACCATCCAAACC |
| *Gapdh* | Forward  Reverse | GGGTGTGAACCACGAGAAAT  ACTGTGGTCATGAGCCCTTC |
| - Mouse genes for RAW264.7 polarization | | |
| *Tgf-β* | Forward  Reverse | CTGCTGACCCCCACTGATAC  AGCCCTGTATTCCGTCTCCT |
| *Cd206* | Forward  Reverse | GACAGACCCAACGGCTTACA  GGTCACAAAACTTCAACCGGA |
| *Arg1* | Forward  Reverse | CAAGCCAAAGCCCATAGAGATT  CATTGGCTTTTCCCACAGACC |
| *iNos* | Forward  Reverse | TCCTCAGGCTTGGGTCTTGT  ATCCTGTGTTGTTGGGCTGG |
| *Tnf-α* | Forward  Reverse | AACAAGGAGGAGAAGTTCCCAAA  CTCCTCCGCTTGGTGGTTT |
| *Cd86* | Forward  Reverse | CTTACGGAAGCACCCACGAT  TGTAAATGGGCACGGCAGAT |
| *Gapdh* | Forward  Reverse | AGTGCCAGCCTCGTCTCATA  GGTAACCAGGCGTCCGATAC |
